# Accounting for spatial and environmental sampling bias in a species distribution model of *Aedes vexans (Diptera: Culicidae)*

**DOI:** 10.1038/s41598-026-61202-5

**Published:** 2026-07-29

**Authors:** Peter Pothmann, Helge Kampen, Doreen Werner, Hans-Hermann Thulke

**Affiliations:** 1 Biodiversity of Aquatic and Semiaquatic Landscape Features, Leibniz Centre for Agricultural Landscape Research, Müncheberg, Germany; 2 Department of Ecological Modelling, Helmholtz-Centre for Environmental Research GmbH, Leipzig, Germany; 3 Department of Forest Science, Dresden University of Technology, Tharandt, Germany; 4 Institute of Infectology, Friedrich-Loeffler-Institut, Greifswald, Germany

**Keywords:** Presence-only modelling, Habitat suitability mapping, Bias correction, Citizen science data, Ecological niche modelling, Ecology, Ecology, Environmental sciences

## Abstract

This study evaluates the potential of species distribution models to predict habitat suitability for the floodplain mosquito *Aedes vexans*, with a particular focus on ephemeral breeding habitats such as flood-prone areas. These habitats are essential for oviposition, yet have not been explicitly incorporated as predictors in existing modelling approaches. Flood hazard was included as an environmental predictor to represent the species’ dependence on transient water bodies. However, initial model runs showed only a marginal contribution of this predictor to overall model performance. We demonstrate that this weak contribution was not due to the ecological irrelevance of ephemeral habitats, but instead resulted from strong sampling biases in the occurrence data, which were predominantly concentrated in urban environments. To address this issue, we applied spatial thinning and environmental filtering to reduce spatial clustering and the overrepresentation of environmentally similar regions. Using Maxent, we compared candidate models with and without bias correction across multiple environmentally stratified test datasets. Our results show that correcting for sampling bias improved model accuracy more than the inclusion of an additional habitat-relevant predictor alone. The final model produced more reliable suitability predictions, particularly in flood-prone and previously under-sampled areas.

## Introduction

Mosquitoes are important vectors of a wide range of pathogens and represent a considerable public health risk. The globally distributed mosquito species *Aedes vexans*, for example, is a competent vector of several arboviruses of medical and veterinary relevance, including West Nile, Rift Valley fever and Zika viruses^1,2^. In Germany, autochthonously acquired infections with West Nile virus have been reported annually between 2018 and 2025, affecting both humans and animals^3^. Understanding the spatial distribution of this species is therefore of high public, veterinary, and ecological interest.

*Aedes vexans* lays its eggs in moist soil near water bodies. These eggs can remain dormant for several years, with larvae only hatching when eggs are flooded by rising water levels^4^. Following an extreme flood event, the concurrent inundation of egg banks, combined with the formation of temporary water bodies, can lead to a mass emergence of adult mosquitoes.

To predict the ecological niche and spatial distribution of species such as *Aedes vexans*, researchers often rely on species distribution models. These models statistically relate species occurrence data to environmental variables in order to estimate the suitability of habitats across geographic space. A key determinant of model accuracy and ecological validity is the choice of environmental variables included in the model. Although high statistical accuracy can sometimes be achieved using variables that are only weakly or indirectly related to the species’ biology, such models often lack ecological interpretability^5^.

Hence, in the case of *Aedes vexans*, habitat suitability should be informed not only by areas regularly influenced by seasonal spring floods, but also by areas that become suitable only after extreme and ephemeral flood events. A previous review of all published species distribution models for *Aedes vexans* showed that none explicitly incorporated flood dynamics as a habitat-defining variable.^6^. In Germany, the CulBase database provides a comprehensive set of physical specimen occurrence records for *Aedes vexans*, compiling data from both the citizen science project *Mückenatlas* and professional monitoring programs^7^. Previous modelling efforts have utilized CulBase data to generate species distribution models for *Aedes vexans*, but without accounting for the sampling biases inherent in opportunistic and unevenly timed observations^8^. Such biases arise because citizen scientists report opportunistically, and professional surveys are irregularly scheduled depending on funding and other resources^9^. Consequently, the ecological contexts of records are unevenly represented: stable seasonal niches are disproportionately captured, while transient habitat conditions following extreme flood events remain underrepresented. Using these occurrence data together with environmental predictors without correcting for sampling bias risks models learning persistent background conditions rather than the drivers of episodic population dynamics, leading to biased response curves, and ultimately misleading predictions in environments strongly shaped by rare events. The aim of our study was to address this gap by developing a species distribution model for *Aedes vexans* in Germany that improves on existing approaches in two key ways: first, by explicitly incorporating a variable representing areas potentially affected by extreme and ephemeral flood events, and second, by reducing the sampling bias towards stable seasonal niches inherent in opportunistic and unevenly timed occurrence records. To this end, we used occurrence data from the German CulBase database^7^ and applied the Maxent algorithm^10^ to build the distribution model. We then evaluated whether inclusion of flood-prone areas, combined with bias correction, improves the *Aedes vexans* distribution model.

## Results

Maxent is among the best-performing species distribution modelling techniques across a wide range of taxa and environmental contexts^11,12^. Furthermore, it is the most commonly applied method in existing distribution models for *Aedes vexans*^6^. The algorithm Maxent requires environmental variables and occurrence points as input data. We used environmental variables derived from existing distribution models for *Aedes vexans*, focusing on predictors that were both influential in shaping model predictions and ecologically meaningful for the species^6^. The selected environmental input variables are listed in Table 4. We expanded the predictor set by incorporating the previously unused variable on areas potentially affected by extreme flood events^13^. The occurrence points were observational records from the CulBase database, which compiles mosquito data across multiple species, collected both through the citizen science project *Mückenatlas* and professional monitoring programs^7^. We extracted all occurrence records of *Aedes vexans*. The CulBase occurrence data is available on Global Biodiversity Information Facility (GBIF)^14^

However, the CulBase data is known to exhibit a sampling bias^9^. Therefore, the modelling process included bias-correction steps using different filtering approaches. We first addressed the spatial sampling bias and then the environmental sampling bias.

### Mitigating spatial sampling bias

We trained a preliminary Maxent model using environmental variables without collinearity and the complete set of CulBase occurrence records for *Aedes vexans* (area under the curve (AUC) = 0.88)^10^. Precipitation showed the highest permutation importance of around 25%. The variable built-up areas exhibited the second highest permutation importance, contributing approximately 20%, while potential flood hazard areas contributed only 0,34%. Other used variables and their respective importance scores are provided in the accompanying data repository (S1)^15^. This was reasonable as citizen science based occurrence records of *Aedes vexans* were over-representing urban areas (sampbias R package^16^ , see S2 & S3 in^15^)^9^.

While urban environments can indeed provide suitable habitat for the species, they are not the only ecologically relevant settings but dominate other ecological habitat descriptors. To further assess the impact of sampling bias towards urban areas, we classified occurrence points into two datasets: urban points and natural points. For each dataset, we trained a separate Maxent model with matching input parameters and tested the performance of the model predictions across both datasets via AUC and five fold cross validation. The outcomes demonstrated distinct differences in model performance between natural and urban habitats for *Aedes vexans*. The model trained on natural occurrence records indicating a good predictive capability within its own environmental setting (AUC 0.79). Interestingly, when tested with urban occurrence data, this model showed an higher AUC of 0.84. This underpins the plausible logic that the environmental conditions captured by the natural habitat model are sufficient to identify the habitat characteristics of the urban records.

**Table 1:** Comparison of AUC values for Maxent models based on natural and urban occurrences of *Aedes vexans*.

Model	Test data	AUC
Natural	Natural	0.793 (± 0.002)
Natural	Urban	0.843 (± 0.005)
Urban	Urban	0.905 (± 0.001)
Urban	Natural	0.753 (± 0.002)

The models were validated using test data from the training environment (Natural or Urban) as well as from the other environment.

In contrast, the model trained on urban occurrence records performed well on urban test data, achieving an AUC of 0.91. However, when applied to natural test data, its AUC dropped to 0.75.

While the natural model exhibited transferability to urban conditions, the urban model struggled when applied to natural areas. We concluded that environmental characteristics captured by urban occurrence points are already represented within natural occurrence points, while the urban points add merely the link with observers. Therefore, urban points were set aside, and modelling was conducted using the natural occurrence records.

### Mitigating environmental sampling bias

Extreme flood zones serve as breeding habitats for *Aedes vexans* only in exceptional years, as water bodies required for oviposition are absent in most years due to a lack of extended flooding. Thus, the potential habitats in flood zones are ephemeral, and natural occurrence records within areas of a historic flood event were limited (number of records in flood zones = 109, number of records outside flood zones = 658)^17^. In addition, most occurrence records come from adult mosquitoes (approx. 99%), which may have dispersed considerable distances from their original breeding sites. Consequently, the data primarily reflect the ecological niche associated with blood-feeding activity rather than that of oviposition sites.

This scarcity leaded to the environmental bias in our occurrence data: natural occurrence points in historically flood-affected areas (AUC = 0.74; see 1) are predicted less accurately than those outside the ephemeral zones (AUC = 0.79).

The high number of non-extreme flood zone records skewed model training and testing, leading to an under-representation of flood-zone habitats and potentially lower suitability scores. To mitigate this bias, we applied an environmental filtering approach to the natural occurrence data^18^. Using this method, we downsampled occurrence records with redundant environmental information. Each environmental variable is partitioned into equally sized bins, and partitions across all variables are combined to identify sections with similar environmental conditions. Assuming that environmental conditions within a given section are comparable, the algorithm randomly selects one record per section^18^. The number of sections can be adjusted by changing the number of partitions per environmental variable. Defining fewer sections results in a greater number of records being discarded. To identify the optimal partitioning, we tested subdivisions ranging from two to nine partitions per environmental variable. For each subdivision, we generated an environment-filtered dataset. The resulting nine datasets, including a dataset without partitioning, were then used to train Maxent models with the same environmental variables as in the natural habitat model. We compared the nine models to determine the partitioning that maximized model performance (Table 2). As evaluation criteria, we used test data representing different niche components and external data: (1) points within flood zones, (2) natural points outside flood zones, (3) urban points outside flood zones, (4) the combination of flood and non-flood points, and (5) external validation data (excluding CulBase data) from the GBIF and the European Centre for Disease Prevention and Control (ECDC)^19,20^. Model performance was assessed using the area under the receiver operating characteristic curve (AUC) and confirmed based on True Skill Statistics (TSS) (TSS results are provided in S4^15^).

**Table 2:** Model performance across different environmental filter parameter settings and testing zones.

Number of partitions for environmental filter	AUC: test in flood zones	AUC: test in natural zones	AUC: test in urban zones	AUC: test in combined zones	AUC: test with external data
2	0.798 (± 0.02) // 10	0.785 (± 0.021) // 7	0.846 (± 0.024) // 22	0.81 (± 0.013) // 9	0.768 (± 0.003) // 0
3	0.818 (± 0.019) // 30	0.795 (± 0.022) // 26	0.851 (± 0.023) // 30	0.821 (± 0.012) // 30	0.776 (± 0.003) // 17
4	0.812 (± 0.019) // 30	0.794 (± 0.021) // 24	0.846 (± 0.024) // 30	0.817 (± 0.012) // 30	0.779 (± 0.003) // 29
5	0.807 (± 0.019) // 30	0.793 (± 0.022) // 22	0.842 (± 0.024) // 30	0.814 (± 0.012) // 30	0.776 (± 0.002) // 23
6	0.805 (± 0.019) // 26	0.793 (± 0.022) // 26	0.841 (± 0.025) // 28	0.813 (± 0.012) // 29	0.777 (± 0.002) // 27
7	0.805 (± 0.019) // 30	0.792 (± 0.022) // 13	0.84 (± 0.025) // 28	0.813 (± 0.012) // 29	0.777 (± 0.002) // 27
8	0.805 (± 0.019) // 27	0.792 (± 0.022) // 13	0.84 (± 0.025) // 26	0.812 (± 0.012) // 28	0.776 (± 0.002) // 9
9	0.804 (± 0.019) // 16	0.792 (± 0.022) // 6	0.84 (± 0.025) // 14	0.812 (± 0.012) // 17	0.776 (± 0.002) // 6
10	0.804 (± 0.019) // 2	0.792 (± 0.022) // 3	0.84 (± 0.025) // 4	0.812 (± 0.012) // 3	0.776 (± 0.002) // 3
All natural records	0.804 (± 0.019)	0.792 (± 0.022)	0.84 (± 0.025)	0.812 (± 0.012)	0.776 (± 0.002)

The table presents mean AUC values (± standard deviation) of 30 model runs for model validation in flood zones, natural zones, urban zones, combined zones, and external datasets. The integer after “//” indicates the number of model runs in which the tested model outperformed the baseline model using the dataset including all natural records.

Overall, models trained with environmental occurrence point filters 3 to 6 outperformed the model based on the unfiltered natural dataset across all niche components and exhibited equal or better performance on external validation data. Filters 3 and 4 showed the highest performance among these, occasionally surpassing each other depending on the evaluation metric. Although filter 4 performed slightly better when evaluated against external datasets, the difference in mean AUC was marginal. In contrast, filter 3 consistently outperformed filter 4 across all other tested environmental gradients and their combinations. Most importantly, filter 3 achieved the highest predictive accuracy within historical flood zones, which are of particular relevance to this study. Therefore, we selected filter 3 for the final model.

Not all environmental filters proved beneficial. The more partitions were defined, the similar became the filtered dataset to the unfiltered version, reducing the intended effect of environmental filtering. On the other hand, filters with very few partitions included too few occurrence points, which negatively impacted model performance due to insufficient data. Although Maxent is known to perform well with relatively few records^10,21^, there appears to be an optimal range—a “sweet spot”—in the number of partitions. Both extremes, too few or too many, led to a decline in model performance in the tested environmental strata.

### Final species distribution model

**Fig. 1:**
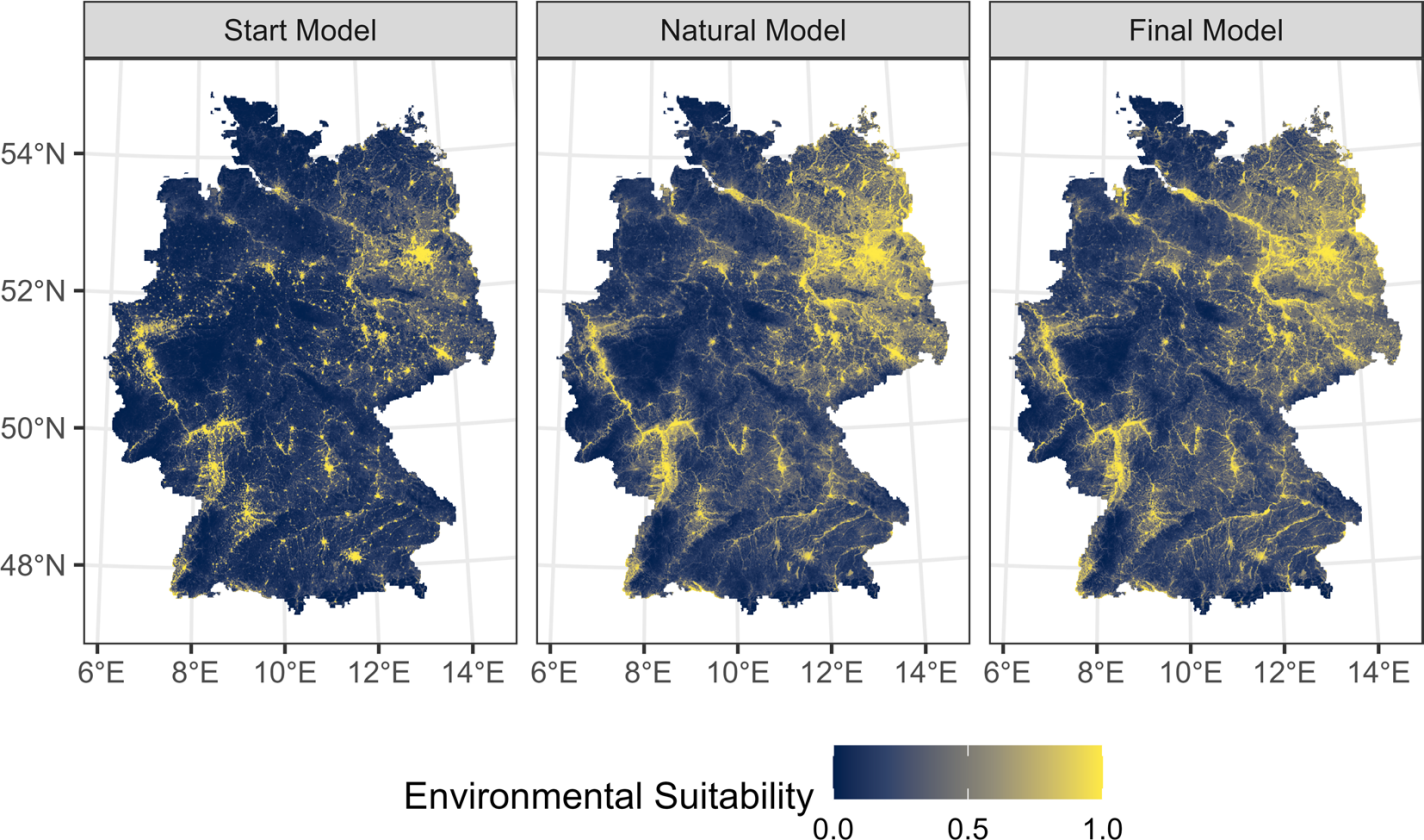
Comparison of environmental suitability predictions of the urban, natural, and final model. The maps show predicted habitat suitability for *Aedes vexans* across Germany, with yellow indicating high suitability and dark blue indicating low suitability. The start model was trained using all available occurrence points, including both natural and urban areas. The natural model used only non-urban (natural) occurrence points. The final model was based on an environmentally filtered subset of the natural occurrence data.

We developed our final species distribution model using the environmentally filtered (partition 3) occurrence data. The internal validation, based on occurrence records retained by the natural filter with three partitions, yielded an AUC of 0.79. This value reflected the model’s capacity to discriminate between presence and background points within the same subset of data that was used for training, thereby providing a measure of internal consistency. External validation, in contrast, was performed using occurrence records independent of the CulBase database. Here, the AUC of 0.77 showed only a slight reduction compared to the internal validation, indicating that the model generalized well beyond the citizen science and monitoring data that formed the training base. The highest performance was obtained when testing the model against natural occurrence records that were not included in the filtered training subset, that is, additional records from CulBase outside the three partition filter. For this test set, the AUC reached 0.79, suggesting that the model not only captures the environmental conditions represented in the training data but also reliably identifies broader ecological patterns across the species’ range with the same performance. Figure 1 compares the environmental suitability predictions of the start model using all occurrence records, the natural model using all natural occurrence records and the final model, trained with the three partition environmental filtered dataset.

**Fig. 2:**
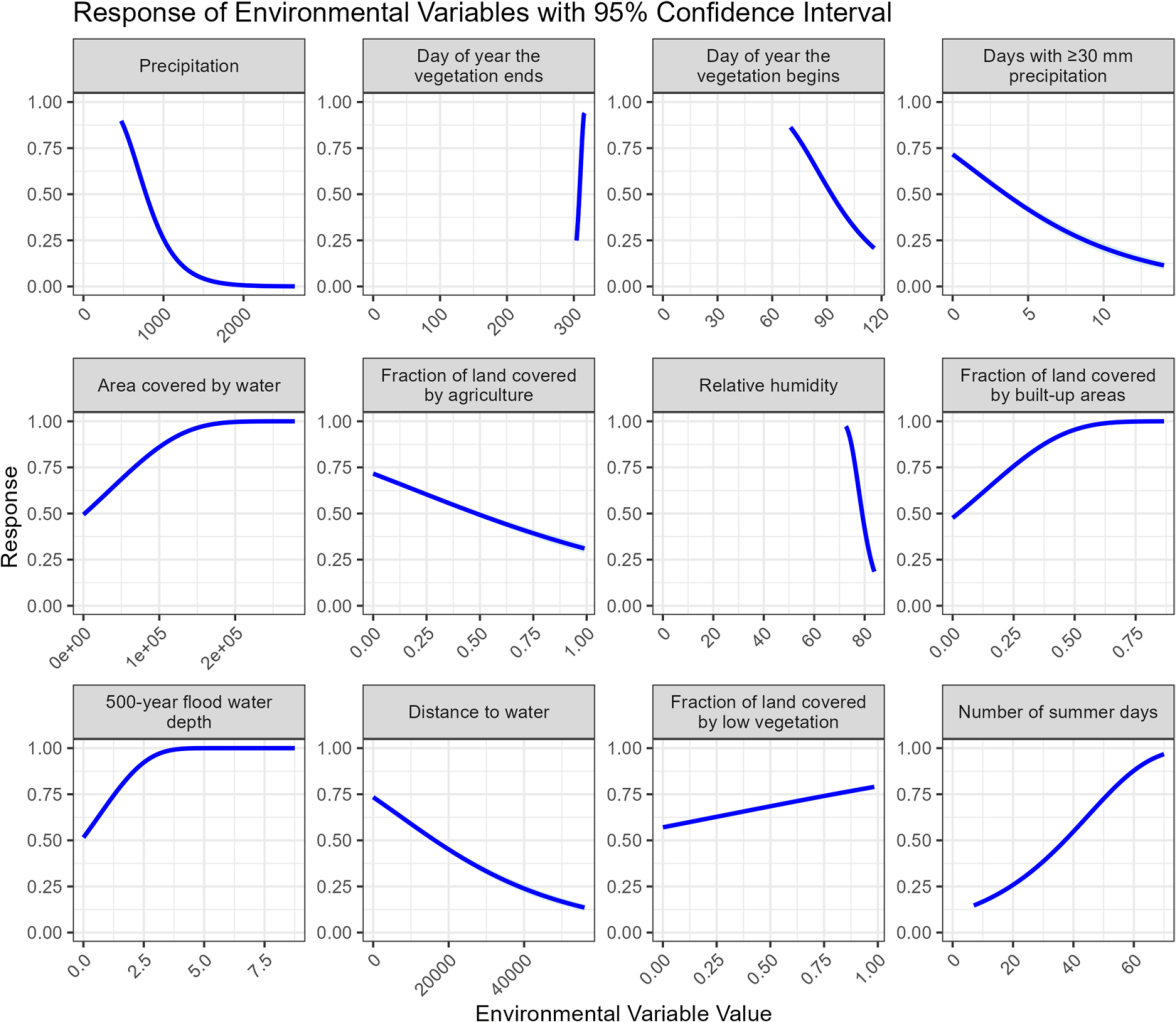
Response curves of environmental predictors. This figure presents response curves illustrating the influence of environmental variables on the predicted habitat suitability of *Aedes vexans*. The y-axis represents the predicted suitability ranging from 0 to 1, while the x-axis shows the values of each environmental variable. The blue lines depict the mean model response and the shaded areas represent the 95% confidence interval across cross-validation runs.

The final model’s variable importance analysis (Table 3) highlighted precipitation as the most influential predictor (38.36 ± 2.09), with a strong negative response: habitat suitability declined sharply as precipitation increased (Fig. 2). Similarly, the number of days with *\ge*30 mm precipitation (6.98 ± 1.32) showed a negative effect, indicating that persistently wet regions with frequent heavy rainfall were less suitable for *Aedes vexans*.

Phenological variables also played a major role. The day of year the vegetation begins (12.20 ± 0.93) showed a negative relationship, pointing to higher suitability where the growing season starts earlier, while the day of year the vegetation ends (18.64 ± 2.47) displayed a positive slope, suggesting a preference for habitats with a later end of the season. Land cover variables contributed to habitat differentiation as well. The fraction of land covered by agriculture (4.40 ± 0.81) showed a negative response. The fraction of land covered by built-up areas (3.98 ± 0.28) exhibited a saturating positive effect, indicating that moderate levels of urbanization may support occurrences, but higher values did not further increase suitability. The fraction of land covered by low vegetation (0.54 ± 0.17) contributed only marginally with a weakly positive slope. The number of summer days (0.24 ± 0.13) had negligible importance in the final model. Hydrological conditions were also important: both the area covered by water (5.28 ± 0.41) and the 500-year flood water depth (3.68 ± 0.60) displayed positive, saturating responses, consistent with the species’ preference for episodic floodplain habitats. The distance to water (1.36 ± 0.55) showed a negative response, indicating that suitability declines with increasing distance from water bodies. Together, these results emphasise that *Aedes vexans* occurrence and development relies on moderate precipitation, early and extended vegetation periods, and episodic floodplain habitats, while areas with frequent heavy rainfall, extensive agricultural cover and long distance to water were less suitable.

**Table 3:** Variable importance of the final species distribution model for *Aedes vexans*.

Environmental variable	Permutation importance	SD
Precipitation	38.36	2.091
Day of year the vegetation ends	18.64	2.472
Day of year the vegetation begins	12.20	0.927
Number of days with *\ge*30 mm precipitation	6.98	1.318
Area covered by water	5.28	0.409
Fraction of land covered by agriculture	4.40	0.812
Relative humidity	4.34	0.733
Fraction of land covered by built-up areas	3.98	0.277
500-year flood water depth	3.68	0.597
Distance to water	1.36	0.550
Fraction of land covered by low vegetation	0.54	0.167
Number of summer days	0.24	0.134

The table presents the mean permutation importance and standard deviation (SD) for each environmental variable, reflecting their contribution to the model’s prediction of habitat suitability.

## Discussion

A central objective of this study was to improve species distribution modelling for *Aedes vexans* by explicitly accounting for ephemeral flood-driven habitats while reducing sampling bias inherent in opportunistic occurrence data. Our results show that the effects of sampling bias outweigh those of ecological predictors, strongly shaping model outcomes. In line with previous studies, uncorrected occurrence data obscure ecologically meaningful patterns^22–24^. In our case, removing urban occurrence records led to a substantial improvement in model performance, exceeding the gains achieved through environmental filtering alone. This finding confirms earlier work emphasizing the importance of bias correction^25–28^ and environmental filtering^18,29^.

Our analysis also provides insight into the interpretation of apparent urban signals in species distribution models. The strong predictive performance of the model trained on natural occurrence records when applied to urban test records suggests that *Aedes vexans* does not fundamentally alter its habitat use in urban environments. Rather, apparent urban preferences in uncorrected models largely reflect sampling bias, where the model captures the spatial structure of sampling effort rather than the species’ ecological niche. This highlights a key challenge in modelling widely distributed and opportunistically sampled species^30–32^.

This is further illustrated by comparison with Wieland et al.^8^, who used the same CulBase occurrence dataset to develop a species distribution model for *Aedes vexans* without applying any sampling bias correction. Their approach combined a climate-based and land-use-based model. The land-use model was strongly influenced by urban structures. In contrast, in our approach we did not separate the climatic and land cover niches but observed a substantially reduced importance of urban areas after bias correction.

However, reducing sampling bias by excluding urban records comes at a cost. In our dataset, approximately two thirds of all occurrence points were located in urban areas, resulting in a substantial reduction in sample size. Although Maxent can perform robustly with limited presence data^10,21^, this approach depends on retaining a sufficient number of ecologically representative records. Thus, a trade-off emerges between bias reduction and data availability. The inclusion of a flood-related predictor contributed to our final model, despite the relatively limited extent of flood-prone areas in Germany. In a recent Netherlands-based model by Dellar et al.^33^, a flood hazard layer was included and had a variable importance of approximately 8%. It is the first publication that included such a layer. Netherlands has a larger proportion of low-lying areas prone to flooding than Germany^13^. As a result, the flood hazard layer in their study provides broader spatial coverage and contributes meaningful variation across more occurrence records. In contrast, many occurrence points fall outside mapped flood-prone areas in Germany and are thus assigned zero values, potentially limiting the overall explanatory power of this variable in our final model (2.96%). The discrepancy in the spatial informativeness of the flood layer may explain its higher importance in the Netherlands-based model.

The European-scale model by Versteirt et al.^34^ predicted only moderate environmental suitability for *Aedes vexans* in Germany, with the lowest values reported for eastern Germany and the Upper Rhine Valley, two regions that our model identifies as highly suitable areas. Dellar et al.^33^ observed that the model developed by Versteirt et al. tends to overestimate habitat suitability in the Netherlands. Regarding Germany, however, the comparison with our results reveals an opposite pattern: areas that our model identifies as highly suitable are often predicted as unsuitable by Versteirt et al.’s model, while regions we classify as having low suitability are frequently predicted as highly suitable by Versteirt et al.

The discrepancy between model predictions is likely due to the coarse spatial resolution and limited occurrence data used in Versteirt et al.’s study. Notably, no occurrence records were available from Germany or the Netherlands at the time of Versteirt et al.’s modelling, meaning that suitability in these regions was extrapolated from presence points in other countries. Their model therefore produced uncertain predictions in the Netherlands and Germany. It overestimated habitat suitability in some areas and underestimated it in others. This highlights the limitations of projecting species distributions into regions without occurrence data^35,36^.

The predicted abundance of *Aedes vexans* from two European Food Safety Authority (EFSA) reports show notable differences compared to our model^37,38^. In the EFSA models, most of Germany is predicted to have low *Aedes vexans* abundance. Areas of moderate abundance are mainly restricted to the Rhine River corridor and the German–Polish border region, while other major river systems are predicted to have low abundance. In contrast, our model identifies several additional major river systems across Germany as highly suitable. However, both models consistently predict lower suitability in northern Germany and in regions east of the Rhine for *Aedes vexans*. Similar to the model by Versteirt et al.^34^, the EFSA products likely overestimate habitat suitability. The models were generated without *Aedes vexans* occurrence data from Germany, meaning suitability estimates were extrapolated from other regions.

Across all discussed studies, precipitation and temperature, or derived variables closely correlated with them, emerge as the most important predictors for the distribution of *Aedes vexans*. This pattern does not only apply to models from Germany, Netherlands and Europe in general but also to studies conducted in other regions^6^.

Several limitations of this study should be acknowledged. First, the occurrence data used for model training were drawn exclusively from the CulBase database, which aggregates records from both citizen science contributions and expert monitoring. We do not account for potential temporal shifts in the ecological niche of *Aedes vexans* over the observation period, as occurrences were treated as temporally homogeneous. Given that climate conditions have changed over the 2011–2023 collection period, this assumption may introduce uncertainty into the model predictions. Furthermore, the dataset contains only a very limited number of records from aquatic life stages—one egg and 19 larval records compared to 6402 adult records—which may bias the model towards habitat conditions favourable for adult mosquitoes rather than capturing the full range of ecologically relevant conditions, particularly those associated with oviposition and larval development. This study relied exclusively on Maxent for species distribution modelling. No alternative algorithms were tested, nor was an ensemble modelling approach considered, in which predictions from multiple algorithms are combined to reduce model-specific uncertainty. Different algorithms may capture non-linear response shapes or interaction effects that Maxent with restricted feature classes cannot represent. In this study, Maxent was further constrained to linear feature classes only, which limits the complexity of the fitted response curves and may prevent the model from capturing threshold effects or unimodal responses *Aedes vexans*. Finally, the study is geographically restricted to Germany, and the transferability of the resulting suitability maps to neighbouring regions or future climate scenarios has not been assessed. Caution is therefore warranted when extrapolating these results beyond the spatial and temporal scope of the training data. In summary, our study demonstrates that explicitly incorporating flood-prone areas and correcting for sampling bias substantially improves the ecological relevance of species distribution models for *Aedes vexans*. These findings highlight the importance of accounting for transient habitat conditions and sampling biases when modelling species with environmentally driven distributions. The resulting suitability maps can directly support public health agencies in prioritising surveillance, targeting vector control measures, and improving disease prevention planning. Finally, our approach illustrates the broader value of embedding species-specific ecological knowledge into SDMs.

## Methods

For our species distribution modelling, we used Maxent^10^. A single set of 10,000 randomly generated background points was created across the study area and used for all models. Due to the large number of models tested, variating the regularization parameter in Maxent could mask changes in occurrence data. Therefore, we set the regularization parameter to 1 and restricted the feature classes to linear terms, which reduces model complexity and limits the tendency to overfit. We used the standard 500 iterations for all models except the final one. For the final model, we applied a genetic algorithm to optimize model quality by adjusting both the regularization parameter and the number of iterations.

### Environmental variables

All environmental predictors used in this study are listed in Table 4. Analyses were conducted at a spatial resolution of 1 km$^2$, using gridded mean temperature data from the German Weather Service (DWD) as a reference grid^39^. All other variables were resampled and aligned to this template to ensure spatial consistency. We chose a 1 km$^2$ resolution as it was the coarsest common resolution across all environmental datasets. To maintain consistency and avoid introducing artificial detail through downscaling, all variables were upscaled to this common grid. Climate-related variables represent the period 1991–2020, with minor variations depending on data availability.

Variables related to surface water included both static components without the aggregation of multiple time steps: current water bodies were derived from OpenStreetMap (OSM)^40^, while flood-prone areas were represented using a flood hazard product^13^. Land-use and land-cover information was obtained from the Mundialis raster product^41^, which provides high spatial resolution (10 m) and a thematically suitable classification, avoiding the need for extensive reclassification. As an alternative, CORINE data were considered but not used due to their coarser spatial resolution and more complex class structure.

Detailed processing steps, data sources, and metadata for all variables are provided in Table S5 in the associated data repository.

**Table 4:** Environmental variables used in the analysis with their measures, sources, and rationale for selection.

Environmental variable	Measure and source	Unit	Reason for selection
Temperature	Maximum (DWD)^42^	$^\circ$C	Several studies link temperature to the occurrence of *Aedes vexans*. For example, temperature influences larval development^43^, and adults are often observed in large numbers when temperatures rise above 30$^\circ$C^44^.
	Mean (DWD)^39^	$^\circ$C	
	Minimum (DWD)^45^	$^\circ$C	
	Number of hot days (DWD)^46^	Days	
	Number of summer days (DWD)^47^	Days	
	Number of frost days (DWD)^48^	Days	
	Number of ice days (DWD)^49^	Days	
Humidity	Number of snow cover days (DWD)^50^	Days	There is a positive relationship between humidity and abundance of *Aedes vexans*^51^. Additionally, a strong increase in precipitation can lead to a higher occurrence of *Aedes vexans*^52^.
	Drought index (DWD)^53^	mm/$^\circ$C	
	Precipitation (DWD)^54^	mm	
	Number of days with *\ge*10 mm precipitation (DWD)^55^	Days	
	Number of days with *\ge*20 mm precipitation (DWD)^56^	Days	
	Number of days with *\ge*30 mm precipitation (DWD)^57^	Days	
	Relative humidity (DWD)^58^	%	
Wind	Wind speed (DWD)^59^	m/s	Smaller insects seek shelter at higher wind speeds, which results in fewer blood meals being taken and fewer eggs being laid^60^.
Vegetation	Day of year the vegetation ends (DWD)^61^	Day of year	Adult mosquitoes prefer vegetated landscapes^44^. Dense vegetation is preferred for oviposition^62^.
	Day of year the vegetation begins (DWD)^63^	Day of year	
	Annual mean NDVI (NASA)^64^	Index	
	Annual mean EVI (NASA)^64^	Index	
	Fraction of land covered by forest (mundialis)^41^	%/100	
	Fraction of land covered by low vegetation (mundialis)^41^	%/100	
	Fraction of land covered by built-up areas (mundialis)^41^	%/100	
	Fraction of land covered by bare soil (mundialis)^41^	%/100	
	Fraction of land covered by agriculture (mundialis)^41^	%/100	
Water	Distance to water (OSM)^65^	m	The mosquito species prefers areas with fluctuating water levels to oviposit. When the oxygen level in the water decreases, larvae begin to hatch from their eggs^66^.
	500-year flood water depth (JRC)^13^	m	
	Area covered by water^40^	m$^2$	
Soil	Soil moisture (DWD)^67^	% NFK	The mosquito species prefers areas with a specific soil moisture level for oviposition^62^.

### Environmental variable selection

We applied a data-driven approach to select environmental variables using the varSel function from the R package SDMtune, to reduce the collinearity^68^. The spearman correlation of the variables are displayed in S6^15^. We applied the variable selection function to all models, with the exception for the model trained with environmental filtered occurrences. For these, we fixed the predictor set to that of the model trained on all natural occurrences. This ensures that the environmental filter models are evaluated with an identical set of predictors, allowing a direct comparison of model performance that is not confounded by differences in variable selection. As a result, model differences are driven solely by the altered selection of occurrence records.

The varSel selection algorithm iteratively evaluates all variables, starting with the one that contributes the most to the model. If a variable is highly correlated with others, as determined by the Spearman method with a correlation threshold of above 0.8, a jackknife test is conducted. Among the correlated variables, the one whose removal improves model performance the most is excluded. This process is repeated until only variables remain with a Spearman correlation threshold below 0.8.

### Occurrence data

We analysed all available occurrence records of *Aedes vexans* (n = 6422) from the CulBase database, collected between 2011 and 2023. These presence-only records originated either from the citizen science project *Mückenatlas* (46.14%) or from active mosquito monitoring conducted by experts (53.75%). For seven records, the sampling source was unavailable.

The *Mückenatlas* is a nationwide citizen science project in which participants collect mosquitoes and submit the physical specimens to the project team for identification. Similarly, specimens collected during expert-based monitoring programmes are physically examined and identified to species level. For each record, associated metadata such as collection location, sampling date, and capture method are stored in the CulBase database.

The occurrence data comprised records from different life stages of *Aedes vexans*, including eggs (n = 1), larvae (n = 19), and adults (n = 6402), all of which were considered species occurrences in the modelling process. Further details on the collection, validation, identification, and management of CulBase records, including data from the *Mückenatlas* citizen science project and expert monitoring programmes, are provided by Werner et al. (2020)^7^. We removed duplicate coordinates, points located in pixels with no values for one or more environmental variables, and points that overlapped within the same raster cells. This filtering resulted in a final CulBase dataset of 2355 occurrence records. All CulBase occurrence records can be found on GBIF^14^.

We additionally included external presence-only *Aedes vexans* occurrence records (no CulBase records) with geographic coordinates in Germany obtained from GBIF on 19 December 2024 (n = 39), as well as records from the ECDC (n = 3)^19,20^, which were used exclusively for model testing.

We assumed that the ecological niche of *Aedes vexans* has remained relatively stable throughout the observation period of the occurrence data. Consequently, occurrences were not distinguished based on time. Therefore, we do not account for potential temporal biases in the dataset^9^.

### Model evaluation

We evaluated model performance using the AUC of the ROC curve. AUC quantifies a model’s ability to discriminate between presences and background points, with values ranging from 0.5 (random prediction) to 1 (perfect discrimination)^69^. While AUC has known limitations it still has several properties that make it well suited for our modelling context^70,71^. First, AUC is threshold-independent: it evaluates the full continuous range of Maxent suitability scores without requiring an arbitrary conversion to binary presence–absence predictions^72,73^. Second, AUC is prevalence-insensitive, meaning it is not affected by imbalances between presence records and background points^74^. Third, Maxent is specifically designed for comparison of presences against background samples, which directly matches the data structure produced by Maxent^10,11^. The AUC metric was calculated using the entire background dataset. To assess the robustness of our model comparisons, we additionally evaluated the key results (Table 2) using TSS as an alternative metric (Table S4)^15^. Despite known limitations of TSS in presence-background settings, in particular the need to treat background points as true absences, which violates the metric’s assumptions, our results show that rankings and conclusions based on AUC were consistent with those obtained using TSS. This indicates that model selection was not driven by the choice of performance metric.

### Sampling bias reduction towards urban areas

We quantified sampling bias for *Aedes vexans* using the sampbias package in two separate analyses: first with all available CulBase occurrence records (n = 2788), and second with the final environmentally filtered dataset (n = 625). We considered the CORINE land-use classes continuous urban fabric, discontinuous urban fabric, water courses, and water bodies as potential drivers of sampling bias. The results of these analyses are provided in the supplementary material (S2 & S3;^15^). In addition, we fitted a Maxent model using all CulBase occurrences to evaluate the variable importance of urban land-use classes.

We assessed the impact of excluding urban areas from the analysis. Occurrence points intersecting CORINE classes continuous urban fabric and discontinuous urban fabric^75^ were classified as urban, with all remaining points designated as natural, comprising 1560 urban and 767 natural points.

Subsequently, separate models were developed for the urban and natural occurrence subsets. Model performance was evaluated via five fold cross validation, and each model was further tested using occurrence points from the opposite habitat category to assess prediction robustness across environments.

### Environmental sampling bias reduction

We account for the environmental sampling bias by using an environmental filtering procedure following^18^. The value range of each environmental variable was extracted and discretized into equal partitions. Then each occurrence point was assigned by its position within the multi-dimensional environmental space. Duplicate positions, indicating environmental redundancy, were removed by retaining randomly a single representative point. We tested nine resolutions (k=2,...,10 partitions per variable) of the filter. The single-interval setting was excluded as it would have reduced the dataset to a single point.

We created a Maxent model for every of the nine environmental filtered datasets. We trained the models using the environmental variables that were selected when creating the natural model. The models were validated using cross-validation with test datasets representing different environmental conditions.

For this purpose, occurrence records were stratified and organized into several test datasets. Table 5 summarizes the different categories and how the datasets were constructed. Model performance was evaluated using AUC for each test dataset. The performance of models incorporating environmental filtering was compared to a baseline model trained on occurrence records from natural areas.

**Table 5:** Environmental test datasets used for model evaluation.

Test dataset	Description/construction
Flood zones	Records located within historical flood areas were identified through spatial intersection with flood extent data from the Copernicus Emergency Management Service, covering all flood events from 2013 to 2024.^17^. A random 20% subset of records was used.
Urban areas	Records outside flood zones located in urban areas, identified using CORINE land-cover classes continuous urban fabric and discontinuous urban fabric. Records of this group were only used for testing. Randomly sampled to match the number of flood-zone test records.
Natural areas	Records outside flood zones in natural areas, defined by subtracting urban records from the total dataset. Randomly sampled to match the number of flood-zone test records.
Combined dataset	Dataset combining all three environmental contexts (flood, urban, natural).
External dataset	Independent occurrence records from GBIF and ECDC^19,20^, used solely for testing.

For each test condition, 30 replicate datasets were generated by randomly sampling occurrence records.

### Identification of the best performing environmental filtered occurrence data set

Among the tested environmental filtering strategies, the filter with three partitions (thereafter filter 3) and four partitions (thereafter filter 4) demonstrated the best overall performance. While the filter with four partitions showed slightly higher mean AUC values when evaluated against external validation datasets, the differences were marginal. Filter 3, on the other hand, consistently yielded higher predictive accuracy across all internally tested environmental gradients and gradient combinations. Furthermore, filter 3 achieved the highest AUC values within the flood zone evaluation subset, which represented a core focus of this study. Therefore, filter 3 was selected for the final model implementation.

### Final model

We developed a final Maxent model, using the environmentally filtered occurrence dataset with three partitions. We used five fold cross validation to evaluate model performance. Predictor variables were re-selected for the final model using the varSel function following^68^, so that the model is based on an independent variable selection step. Additionally, variables contributing less than 15% to the permutation importance were evaluated using a jackknife test to assess their impact on model performance. Variables whose exclusion led to a decrease in AUC were removed. To further optimize model performance, we tuned the regularization multiplier and the maximum number of iterations, using a genetic algorithm provided by the SDMtune R package^68^. We tested regularization values from 1 to 5 in increments of 0.1 and the maximum number of iterations from 100 to 1000 in increments of 100. The highest predictive performance was achieved with a regularization score of 1.3 and 1300 iterations.

## Supplementary Information

Supplementary Information.

## Data Availability

All primary data used in this study are publicly available and can be accessed through the references provided in the manuscript. The occurrence data provided by the European Centre for Disease Prevention and Control, are available upon request from the respective institution. The associated zenodo data repository contains supplementary materials related to this study, including detailed variable importance metrics, model performance across different model configurations, and the geodata of the spatial predictions. The supplementary materials associated with this article are available in the Zenodo repository at 10.5281/zenodo.15090542.
