# Supplementary material for: Accounting for spatial and environmental sampling bias in a species distribution model of Aedes vexans (Diptera: Culicidae)
Source: Sci Rep. 2026 Jul 29;16:23622. doi: 10.1038/s41598-026-61202-5 (PMC13424090; doi:10.1038/s41598-026-61202-5)
Supplement: Supplementary file 1 — Supplementary Information. [file 41598_2026_61202_MOESM1_ESM.pdf]

Supplementary Materials: Disentangling Spatial and Environmental Sampling Bias in Species Occurrence Data: A Species Distribution Model for the Mosquito *Aedes vexans*

Peter Pothmann[1,2,3,*], Doreen Werner[1], Helge Kampen[4], Hans-Hermann Thulke[2]

[1]Leibniz Centre for Agricultural Landscape Research, Germany
[2]Helmholtz-Centre for Environmental Research GmbH, Germany
[3]Technische Universität Dresden, Germany
[4]Friedrich-Loeffler-Institut, Germany
[*]peter.pothmann@zalf.de

Supplementary Table S1: Example Table Title

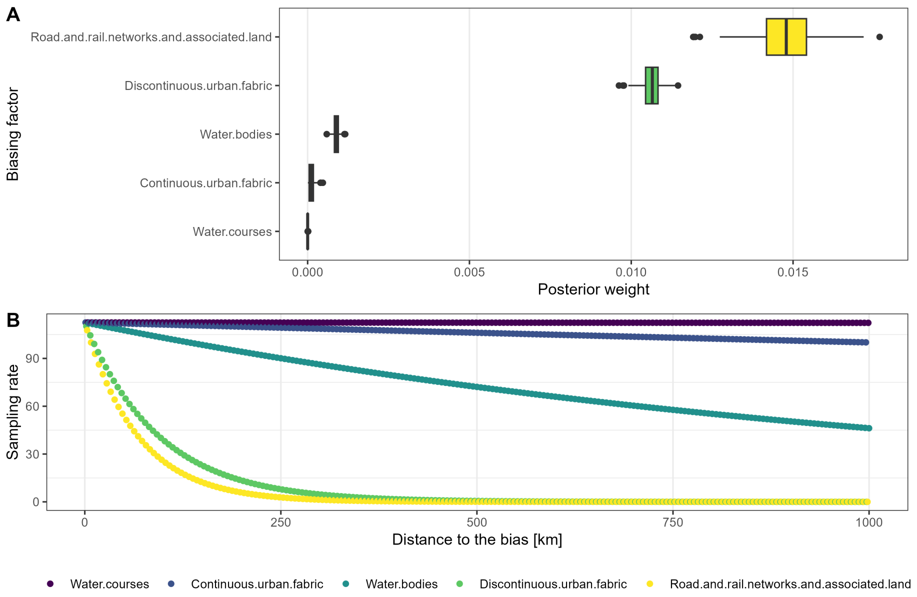

Figure 1: Effects of different landscape features on spatial sampling bias before environmental filtering. (A) Posterior weights of four landscape features derived from a bias estimation model, indicating their relative contribution to spatial sampling bias. Higher weights suggest a stronger association with observer presence. (B) Corresponding sampling rate functions showing how sampling effort decreases with increasing distance from each biasing feature. Created using the *sampbias* R package

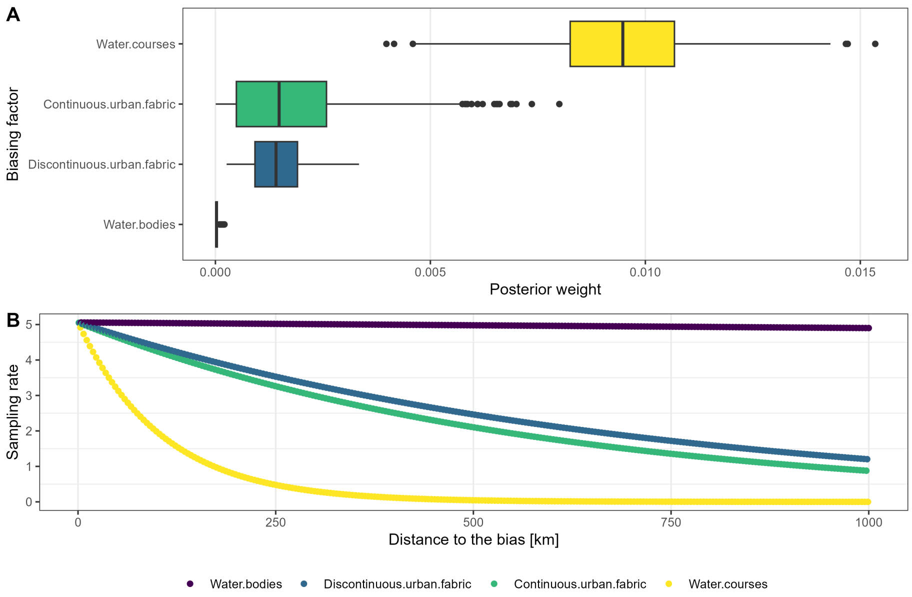

Figure 2: Effects of different landscape features on spatial sampling bias after environmental filtering. (A) Posterior weights of four landscape features derived from a bias estimation model, indicating their relative contribution to spatial sampling bias. Higher weights suggest a stronger association with observer presence. (B) Corresponding sampling rate functions showing how sampling effort decreases with increasing distance from each biasing feature. Created using the *sampbias* R package.

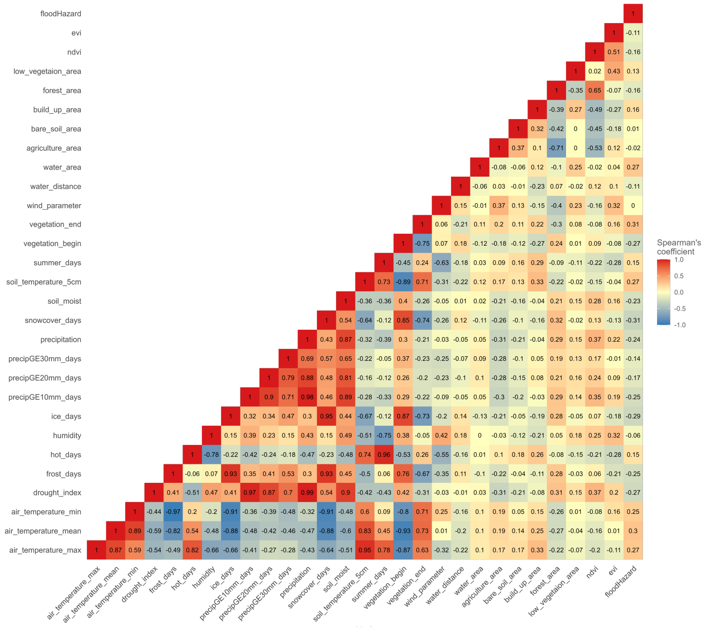

Figure 3: Plot of the Pearson correlation matrix displaying pairwise relationships between variables. Colors indicate the strength and direction of correlations, with values ranging from -1 (perfect negative) to 1 (perfect positive).

Table 1: Example table caption for TSS-based performance.

Environmental filter parameter	TSS - Test in Flood zones	TSS - Test in normal natural zones	TSS - Test in normal urban zones	TSS - Test both zones	TSS - Test external data
2	0.477 (± 0.042) // 13	0.462 (± 0.037) // 7	0.576 (± 0.053) // 28	0.49 (± 0.028) // 15	0.493 (± 0.016) // 21
3	0.508 (± 0.037) // 28	0.48 (± 0.039) // 15	0.564 (± 0.049) // 30	0.5 (± 0.022) // 30	0.5 (± 0.007) // 28
4	0.5 (± 0.035) // 25	0.477 (± 0.034) // 13	0.557 (± 0.051) // 29	0.494 (± 0.023) // 27	0.486 (± 0.005) // 26
5	0.494 (± 0.034) // 23	0.479 (± 0.035) // 17	0.546 (± 0.052) // 27	0.488 (± 0.022) // 21	0.484 (± 0.006) // 30
6	0.493 (± 0.034) // 22	0.479 (± 0.036) // 13	0.542 (± 0.052) // 26	0.486 (± 0.022) // 21	0.482 (± 0.006) // 26
7	0.491 (± 0.034) // 17	0.479 (± 0.036) // 16	0.54 (± 0.052) // 16	0.485 (± 0.021) // 19	0.48 (± 0.006) // 5
8	0.49 (± 0.034) // 14	0.479 (± 0.037) // 14	0.54 (± 0.052) // 21	0.485 (± 0.022) // 15	0.481 (± 0.006) // 10
9	0.49 (± 0.035) // 11	0.479 (± 0.037) // 11	0.54 (± 0.052) // 8	0.485 (± 0.022) // 11	0.48 (± 0.006) // 2
10	0.49 (± 0.035) // 5	0.479 (± 0.037) // 2	0.54 (± 0.052) // 2	0.485 (± 0.022) // 4	0.481 (± 0.006) // 3
Original dataset	0.49 (± 0.035)	0.479 (± 0.037)	0.54 (± 0.052)	0.485 (± 0.022)	0.481 (± 0.006)
